# Quality and reliability of YouTube videos on breast engorgement

**DOI:** 10.1186/s13006-026-00837-6

**Published:** 2026-04-03

**Authors:** Şehma Şen, Meserret Aslan, Ece Nilüfer

**Affiliations:** 1 Nursing (English), İstanbul Atlas University, Anadolu Street, İstanbul, 34408 Türkiye; 2 Midwifery, İstanbul Atlas University, Anadolu Street, İstanbul, 34408 Türkiye

**Keywords:** Breast engorgement, Breastfeeding, DISCERN, GQS, JAMA, Postpartum, YouTube

## Abstract

**Background:**

Breast engorgement is a common and painful condition that can lead to the early cessation of breastfeeding. Many mothers turn to online platforms like YouTube for information and support, but the quality of this content is often unverified. This study aims to evaluate the quality and reliability of YouTube videos concerning breast engorgement.

**Methods:**

A systematic search was conducted on YouTube in June 2025 using six relevant keywords. After excluding videos that were non-English, irrelevant, duplicates, “Shorts,” or “vlogs,” 116 videos were included in the final analysis. Three independent researchers evaluated the videos using the DISCERN instrument, the Journal of the American Medical Association (JAMA) measurement tool, and the Global Quality Score (GQS). Video characteristics and popularity metrics (views, likes, comments) were also collected. Statistical analysis was performed to compare videos based on uploader characteristics.

**Results:**

The overall quality of the videos were moderate, with mean scores of 51.78, 2.25, 3.49 for DISCERN, JAMA, and GQS, respectively. Videos uploaded by healthcare professionals (*n* = 58) had significantly higher quality scores across all instruments (DISCERN: *p* < .001; JAMA: *p* = .015; GQS: *p* < .001), as well as longer durations (*p* = .007) and more likes (*p* = .002), compared to those uploaded by non-healthcare creators. While videos uploaded by healthcare professionals received significantly more likes (*p* = .002), those from non-healthcare sources had significantly higher viewer interaction rates (*p* < .001) and were more popular in terms of views (*p* < .05), highlighting a “quality–popularity paradox”. Correlation analysis revealed strong positive relationships between views and likes (*r* = .705, *p* < .001), and between likes and viewer interaction (*r* = .613, *p* < .001). While quality scores were positively correlated with popularity metrics, these associations were weak (e.g., GQS and views: *r* = .259, *p* = .005). No significant correlation was found between JAMA scores and popularity indicators (*p* > .05).

**Conclusions:**

While YouTube serves as a crucial information resource for mothers experiencing breast engorgement, the most popular videos are often not the most reliable. Therefore, ensuring that mothers receive individualized support in managing conditions like breast engorgement should remain a primary focus. Additionally, strategies to reduce misinformation and the implementation of verification systems for health-related content on platforms like YouTube should be considered.

## Background

The first 12 weeks after birth, known as the “postpartum period” or the “fourth trimester,” is a transitional period of critical importance for the health of the mother and baby, where physical and psychological recovery occurs, the mother-infant bond is strengthened, and care needs increase [1]. ACOG recommends an assessment via face-to-face or telephone contact within the first three weeks after birth and the completion of a comprehensive biopsychosocial evaluation by the 12th postpartum week [2]. In parallel, the World Health Organization (WHO) advises postpartum health check-ups that include both the mother and the newborn on the third day, between the seventh and fourteenth days, and at the sixth week after delivery [3]. During this period, supporting the mother holistically is as crucial for maternal and infant health as maintaining and supporting breastfeeding [4].

Breastfeeding provides significant health benefits for both breastfed infants and breastfeeding mothers [5]. Various health organizations recommend exclusive breastfeeding for the first 6 months of life and continuing for at least one year with the addition of complementary foods, if it is mutually desired by the mother and infant [6, 7]. WHO [8] recommends that all infants be exclusively breastfed for the first six months of life and then continue to receive breast milk with appropriate complementary foods up to two years of age or beyond. Despite this, reported breastfeeding rates at 24 months remain very low, with one cohort study reporting a rate of 2.3% and data from Australia showing less than 3% [9]. This low rate may be associated with the challenges faced by working mothers, as more than half of women with infants under one year are in the labor force, and nearly 75% of companies do not provide adequate lactation support in the workplace [10, 11]. One of the most common reasons for the early cessation of breastfeeding is difficulties related to milk production, particularly the inability or perceived inability to produce enough milk [12, 13]. Breast engorgement is a pathological condition that typically occurs within the first week postpartum, resulting from the overfilling of the breasts with milk due to obstructed milk flow. It manifests with hardness, pain, and tension, often causing discomfort and complicating the breastfeeding process [14–16]. This condition, common among breastfeeding women, may lead to blocked milk ducts and infections such as mastitis. Studies have reported that 52% of mothers experience breast pain 3–7 days after delivery [17], and 33.9% report high-intensity breast pain in the second postpartum week [18]. Moreover, in the early postpartum period, 26% of mothers discontinue exclusive breastfeeding due to issues such as breast engorgement, nipple pain, or cracks [19]. The occurrence of breast engorgement has also been associated with the early introduction of supplementary foods from sources other than breast milk [20].

Although various medical and non-medical treatment methods for the relief and management of breast engorgement have been examined, the number of interventions with proven effectiveness remains limited [21]. Meta-analyses have indicated that cabbage leaves, acupuncture, and acupressure may provide benefits in reducing breast engorgement symptoms among breastfeeding mothers [15, 16]. However, there is still insufficient evidence to recommend any single method for widespread clinical use. In practice, International Board Certified Lactation Consultants (IBCLCs) and Certified Lactation Counselors (CLCs) commonly recommend low-risk and easily applicable methods such as frequent and effective breastfeeding, proper positioning, gentle breast massage, and the application of warm or cold compresses to relieve discomfort, consistent with the guidance of the Academy of Breastfeeding Medicine [22]. The ABM protocol also highlights the importance of early lactation support, adequate milk removal, correct latch, and reassurance to prevent and manage engorgement effectively. Furthermore, education provided by clinical staff in the early postpartum period—focusing on proper breastfeeding techniques, frequent feeding, and practical strategies for managing discomfort—has been shown to reduce breastfeeding problems experienced by mothers [23]. A systematic review and meta-analysis of 22 studies reported that breastfeeding education is effective in reducing breast engorgement and pain and increasing exclusive breastfeeding rates in the first 1–6 weeks postpartum, emphasizing that health professionals should implement comprehensive training programs that combine theoretical knowledge and hands-on practice to maintain this effect [21]. However, access to clinical lactation consultants is often limited, and appointments can be difficult to obtain. As a result, many mothers seek information and practical guidance from online platforms such as YouTube. Online platforms, social media, and informational videos are increasingly becoming common sources of health information, with studies showing a high percentage of users consuming health content on YouTube. In the age where 8 out of 10 internet users can access health-related information online, the influence and power of social media in disseminating such information cannot be ignored [24–26]. Considering that many of the analyzed videos include subtitles and on-screen text, the readability of such materials is important for viewer comprehension and health literacy. The National Institutes of Health, the US Department of Health and Human Services, and the American Medical Association recommend that patient education materials be written below a sixth-grade reading level to ensure accessibility and understanding [26]. Social media platforms, which have recently gained a significant place in individuals’ lives, emerge as supportive and encouraging areas that can have positive or negative effects on breastfeeding and breastfeeding education [27]. In the literature, there are very few studies on the content of breastfeeding education on social media, the accuracy of the shared information, and the profile of the individuals sharing this information [28, 29]. YouTube, the platform that first initiated video sharing in 2005, now receives billions of uploads and visits daily [30]. The social media platform YouTube has significant potential for delivering personalized health education to diverse audiences [31].

In a condition like breast engorgement, where timely and accurate advice is critical for supporting breastfeeding continuation and preventing complications, the quality of online information is paramount. While previous research has evaluated general breastfeeding content on social media, there is a significant lack of objective data focusing specifically on the quality and reliability of information regarding the management of breast engorgement on YouTube. This study seeks to fill this gap by providing a holistic and systematic evaluation of videos dedicated to this specific complication. Unlike broader studies, our research uniquely identifies the ‘quality-popularity paradox’ in breast engorgement content, offering a more nuanced understanding of how uploader characteristics influence both the accuracy and the reach of postpartum health information. Acknowledging this critical need, this study aimed to holistically evaluate the quality of YouTube videos on the topic of breast engorgement using standardized instruments like DISCERN, JAMA, and GQS to establish a robust rationale for clinical guidance in the digital age.

## Methods

Ethical principles were adhered to throughout the study process, and no personally identifiable data was collected. As the research was based solely on the analysis of publicly available YouTube videos, ethics committee approval was not required. Furthermore, informed consent forms were not obtained since no participant data was used and only video content was analyzed.

To evaluate the quality of YouTube videos on breast engorgement, a descriptive cross-sectional content analysis was conducted on the YouTube platform (https://www.youtube.com) in June 2025. The keywords used for the search were: “breast engorgement”, “relief for breast engorgement”, “breastfeeding and engorgement”, “how to manage breast engorgement”, “breast engorgement treatment”, and “painful breastfeeding breasts”. All videos listed after entering the keywords were viewed. A total of 578 videos on this topic were found on YouTube.

**Fig. 1:**
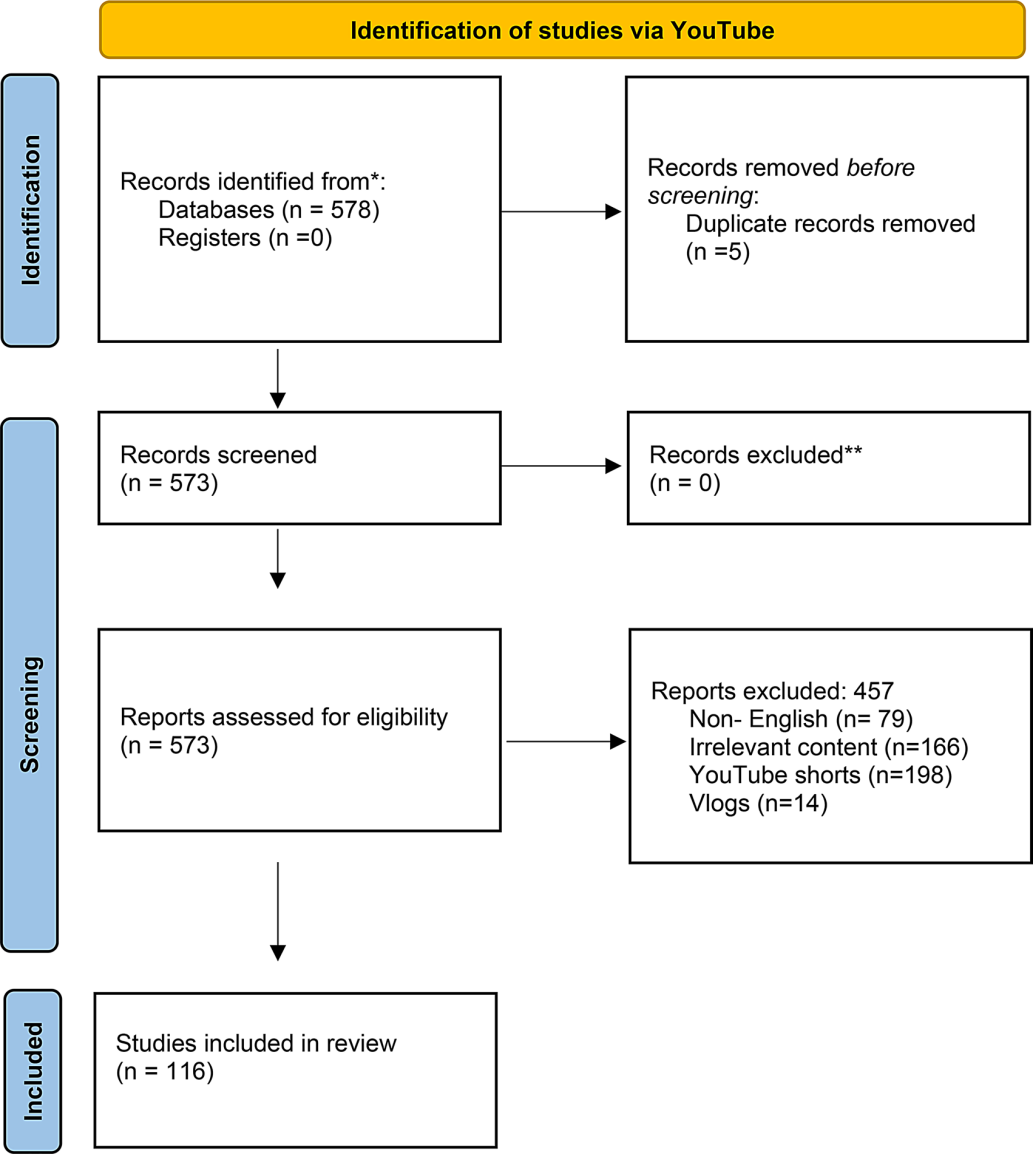
PRISMA flow diagram

Before conducting the search on YouTube, incognito mode was activated in the Google Chrome browser. By doing so, previous user data and browser history were disabled to prevent search results from being influenced by personalized interactions, aiming to obtain more objective results. During the YouTube searches, the platform’s default “relevance” sorting option was used, and a natural search method reflecting user search behavior was adopted without any additional filtering. The videos identified in the preliminary review underwent a systematic screening process. A total of 457 videos were excluded for reasons such as being non-English (*n* = 79), having irrelevant content (*n* = 166), being in “YouTube Shorts” (*n* = 198) or “vlog” (*n* = 14) format. Additionally, 5 duplicate videos—i.e., the same video appearing more than once in different searches—were also eliminated. As a result of this screening process, 116 unique videos that met the research criteria constituted the final sample for the study (Fig. 1).

All evaluations were conducted independently by three researchers – Ş.Ş. (an obstetrics and gynecology nurse), M.A. (a midwife), and E.N. (a midwife) – evaluated the videos based on criteria of reliability and overall quality. In cases of disagreement, a consensus was reached through discussion among the reviewers. During the evaluation process, the DISCERN Score, the Journal of the American Medical Association (JAMA) Score, and the Global Quality Score (GQS), which are standardized measurement tools widely used for the objective analysis of health-related video content, were utilized. These instruments are frequently preferred reliable tools in the literature for assessing the scientific accuracy and informational value of content [24, 32–34].

DISCERN is a validated and reliable measurement tool developed to assess the quality and reliability of health information materials. This tool allows for the objective analysis of the presentation of information, particularly regarding treatment options. DISCERN consists of a total of 16 items, each scored from 1 (poor) to 5 (excellent), resulting in a total score range of 16 to 80. The first eight items focus on content accuracy and reliability, while the ninth to fifteenth items address the clarity and adequacy of treatment options. The sixteenth item involves an overall quality assessment [35]. DISCERN has been used as an effective tool in many previous studies to evaluate elements that constitute information reliability, such as the transparency of evidence and the unbiased presentation of information [36–38].

The Journal of the American Medical Association (JAMA) Score is a four-item quality measurement tool developed to assess the reliability of online health information content. This instrument is based on four fundamental criteria: authorship, citation of sources, currency of information, and disclosure of conflicts of interest. Each criterion is scored as either 0 (not met) or 1 (met), resulting in a total score range from 0 to 4. A higher score indicates that the content is more reliable and transparent [39, 40].

The Global Quality Score (GQS) is a subjective measurement tool used to evaluate the overall quality and educational value of online health content. This score, rated on a five-point Likert scale, is graded from 1 (very poor) to 5 (excellent), considering the content’s clarity and benefit to the user. GQS is particularly preferred for reflecting the overall usefulness and informational value of health-related video and web-based materials from the viewer’s perspective [40].

During the review process, the popularity of each video was evaluated based on the number of views, likes, and video duration. Additionally, it was recorded whether the uploader of the video was a healthcare professional. The scoring process was conducted by three independent reviewers who did not communicate with each other during the evaluation. The viewer interaction rate was calculated by subtracting the number of dislikes from the number of likes, dividing the resulting value by the total number of views, and finally multiplying by 100 [41, 42]. Although YouTube has restricted the public visibility of dislike counts since late 2021, these data were retrieved using the ‘Return YouTube Dislike’ API/plugin (https://returnyoutubedislike.com/). This approach was adopted to ensure a comprehensive assessment of viewer engagement and to maintain the integrity of the interaction ratio, as dislike counts remain a critical indicator of content dissatisfaction in health informatics research.

In order to categorize the videos based on the presence of healthcare providers, we examined how individuals identified themselves within the video content. Those who explicitly introduced themselves as nurses, physicians, or lactation consultants were classified as healthcare providers. Conversely, individuals who did not provide any personal or professional information in the video or on their YouTube channel were categorized as non-healthcare providers. This classification was based solely on self-identification and publicly available channel information.

Statistical evaluations were performed using IBM SPSS Statistics 26.0 (Armonk, NY, USA) software. Descriptive statistics were used to describe the sample; continuous variables were expressed as mean ± standard deviation (SD) and median (minimum–maximum) values, while categorical data were presented as frequencies and percentages. The distribution characteristics of the data were examined using the Kolmogorov–Smirnov test. For the comparison of continuous variables, the Mann–Whitney U test was used for non-normally distributed data, while appropriate parametric tests were applied for normally distributed data. The Spearman correlation coefficient was used to analyze the relationship between variables. In all analyses, the statistical significance level was considered within a 95% confidence interval, and a p-value of < 0.05 was accepted as statistically significant. The kappa coefficient was calculated to determine the consistency among the three independent evaluators; numerical scores were determined by taking the arithmetic mean of the scores from two evaluators.

## Results

Descriptive statistics for the analyzed breast engorgement videos are presented in Table 1. The mean number of views was 116,963.41 ± 847,074.01, and the mean number of likes was 135.62 ± 456.94. The mean duration of the videos was 372.52 ± 307.31 s, and the mean time since release was 43.33 ± 41.89 months. According to the quality assessment tools, the mean DISCERN, JAMA, and GQS scores were 51.78 ± 7.98, 2.25 ± 0.78, and 3.49 ± 0.70, respectively. Based on standard interpretation guidelines—DISCERN (40–59), JAMA (2–3), and GQS (3–4)—these values indicate moderate overall quality. These scores indicate that the overall quality level of the analyzed videos is moderate.

**Table 1:** Descriptive statistics of breast engorgement videos

	Mean ± SD	Median (Minimum–maximum)
Views (n)	116963.41 ± 847074.01	577 (6- 9000000)
Time Since Release (months)	43.33 ± 41.89	36 (1-244)
Likes (n)	135.62 ± 456.94	4.5 (0-3500)
Length (min)	372.52 ± 307.31	295 (32-1898)
Viewer interactions	2.77 ± 19.73	0.42 (-0,40- 212.87)
DISCERN score	51.78 ± 7.98	52 (34.67- 69)
*JAMA* score	2.25 ± 0.78	2.33 (0.67- 4)
GQS	3.49 ± 0.70	3.67 (1.67–4.67)

Abbreviations: GQS, Global Quality Scale; JAMA, Journal of the American Medical Association

**Table 2:** Analysis of videos based on occupation status

	Healthcare (*n* = 58) Mean ± SD	Nonhealthcare (*n* = 58) Mean ± SD	*P* value^a^
Views (n)	28,166.43 ± 81,447.32	205,60.39 ± 1,193,723.97	0.110
Time Since Release (months)	36.7 ± 31.49	49.94 ± 49.59	0.267
Likes (n)	151.74 ± 492.8	119.5 ± 421.74	0.002
Length (min)	438.25 ± 365.98	306.77 ± 218.62	0.007
Viewer interactions	1.25 ± 1.63	4.28 ± 27.89	< 0.001
DISCERN score	54.33 ± 7.42	49.23 ± 7.75	< 0.001
*JAMA* score	2.42 ± 0.75	2.06 ± 0.77	0.015
GQS	3.79 ± 0.52	3.19 ± 0.69	< 0.001

Abbreviations: GQS, Global Quality Scale; JAMA, Journal of the American Medical Association

^a^Mann Whitney U test

Mann-Whitney U test comparisons based on the content creator group (Healthcare, *n* = 58; non-healthcare, *n* = 58) showed statistically significant differences between the groups across various variables (Table 2). The analyses revealed no significant difference between the two groups in terms of video view count (*p*= .110) and time since release (*p*= .267).

In contrast, videos created by healthcare professionals were found to be statistically superior across several metrics. The videos from this group had a significantly higher number of likes (*p*= .002), a longer mean video duration (*p*= .007), and higher scores on all three-quality metrics (DISCERN, *p*< .001; JAMA, *p*= .015; GQS, *p*< .001) compared to the non-healthcare group.

Contrary to this general trend, only the viewer interaction rate was found to be significantly higher in the videos from non-healthcare content creators (4.28 ± 27.89) compared to the healthcare group (1.25 ± 1.63) (*p*< .001). These findings indicate that while videos created by healthcare professionals are of higher quality, longer, and more liked, non-healthcare videos generate a higher viewer interaction rate.

**Table 3:** Correlation between some video parameters and the scoring systems

	*P* or *r* value	Likes	Viewer interactions	JAMA score	GQS	DISCERN score
Views (n)	*r*	0.705	0.061	0.071	0.259	0.143
	*P*	< 0.001	0.516	0.447	0.005	0.126
Time Since Release (months)	*r*	0.386	− 0.064	0.026	0.116	-0.70
	*P*	< 0.001	0.494	0.782	0.213	0.453
Likes (n)	*r*	-	0.613	− 0.040	0.263	-
	*P*	-	< 0.001	0.666	0.004	-
Length (min)	*r*	0.200	0.352	0.065	0.043	0.015
	*P*	0.032	< 0.001	0.488	0.648	0.876
Viewer interactions	*r*	0.613	-	0.071	0.259	0.086
	*P*	< 0.001	-	0.447	0.005	*P* = .356
DISCERN score	*r*	0.105	0.086	0.640	0.552	-
	*P*	0.261	0.356	< 0.001	< 0.001	-
JAMA score	*r*	− 0.040	− 0.064	-	0.519	-
	*P*	0.666	0.493	-	< 0.001	-
GQS	*r*	0.263	0.146	0.519	-	-
	*P*	0.004	0.119	< 0.001	-	-

Abbreviations: GQS, Global Quality Scale; JAMA, Journal of the American Medical Association; r, Spearman correlation coefficient

The results of the Spearman’s rho correlation analysis, conducted to determine the relationships between variables, are presented in Table 3.

When examining the popularity metrics, statistically significant and strong positive correlations were identified between the number of views and the number of likes (*r* = .705, *p* < .001), and between the number of likes and the viewer interaction rate (*r* = .613, *p* < .001). Similarly, the quality assessment tools were also positively and significantly correlated with each other; strong relationships were particularly observed between the DISCERN score and the JAMA score (*r* = .640, *p* < .001) and the GQS (*r* = .552, *p* < .001). Furthermore, there was a positive correlation between the JAMA score and the GQS (*r* = .519, *p* < .001).

When the relationship between content quality and popularity was examined, only weak but statistically significant positive correlations (*r* = .259–0.386, *p* < .05) were observed, indicating that these associations, although significant, were of low practical strength. The Global Quality Score (GQS) showed a statistically significant, yet weak, positive relationship with the number of views (*r* = .259, *p* = .005), the number of likes (*r* = .263, *p* = .004), and the viewer interaction rate (*r* = .259, *p* = .005). In contrast, the JAMA score was found to have no significant relationship with any of the popularity metrics (*p* > .05).

Finally, among the video characteristics, time since release was found to have a significant positive relationship with the number of likes (*r* = .386, *p* < .001), while video length had significant positive relationships with both the number of likes (*r* = .200, *p* = .032) and the viewer engagement rate (*r* = .352, *p* < .001).

## Discussion

This study aimed to investigate the quality and reliability of YouTube videos concerning breast engorgement. The high number of views reached by the examined videos indicates that breast engorgement is a frequently searched topic on YouTube and that new mothers use this platform as a source of information. The existing videos generally aim to help mothers understand and find solutions to their problems by providing information on the causes, symptoms, and management of breast engorgement. Access to accurate information can enable mothers to make more informed decisions during this process. Although videos created by healthcare professionals were found to be of higher quality when evaluated with criteria such as DISCERN, JAMA, and GQS, the most striking finding of our study was a *“quality-popularity paradox.”* The fact that this superiority in content quality does not directly translate to popularity, and that higher viewer interaction is often seen on lower-quality videos, points to a significant challenge for mothers seeking reliable information in a digital landscape where engagement often outweighs scientific accuracy.

In an era where digital platforms are primary sources of health information [24], YouTube has become an easily accessible and commonly used platform for pregnant and postpartum women. While this accessibility is an advantage, the lack of a peer-review or verification process for uploaded content presents a significant public health concern. Videos on YouTube have many advantages, such as being visually appealing, easily accessible, and reaching a wide audience; however, they contain significant problems regarding accuracy and reliability [43]. This reliability issue is further deepened by the platform’s operational mechanism, as YouTube’s algorithms recommend videos based on users’ past data and rank videos from more popular content creators higher, which can create a breeding ground for the spread of health-related misinformation [44]. The finding in this study that breast engorgement videos are generally of moderate and variable quality parallels the findings in the literature that question the reliability of health information on the YouTube platform [34, 45, 46]. Similar findings have been reported in previous studies evaluating the quality of breastfeeding-related content on YouTube. In a study examining 200 videos, only about 18.8% were classified as good or excellent quality, indicating that breastfeeding content on YouTube provides a limited source of accurate information for patients [47]. Another study analyzing 165 videos found that most had moderate reliability and quality, with educational videos and those created by healthcare professionals showing higher quality levels. These findings are consistent with our results, highlighting the variability and limitations of health information on this platform. Furthermore, it was revealed that new mothers need accurate information due to the challenges they face regarding their self-care and newborn care, and they attempt to acquire this information through YouTube, one of the social media platforms [31]. Our analysis reveals that the identity of the content creator lies at the core of this quality variability. Indeed, it was determined that videos created by healthcare professionals had statistically significantly higher scores on all quality-measuring metrics compared to the non-healthcare group. This finding is also consistent with previous research reporting that content produced by qualified experts is more reliable and contains less misinformation [48].

The existing literature supports the “quality-popularity paradox” observed in medical content on YouTube. Research indicates that view count or popularity is not directly correlated with the accuracy or quality level of a video [49]. Indeed, it has been reported that the most-watched videos can often contain less comprehensive or erroneous information [50]. It is suggested that this situation may be related to presentation style rather than informational accuracy; the higher engagement of low-quality videos is often attributed to narrative-focused and emotionally connecting content strategies [32, 51]. Non-healthcare content creators are observed to encourage viewers to comment or share through personal storytelling, simple language, and eye-catching visuals [43]. In contrast, while videos prepared by healthcare professionals score higher in quality assessments, it is suggested that their information-dense, didactic presentations may be perceived as less engaging by the general audience [52]. In this context, presenting information in a way that can connect with the target audience appears to be as critical for effective health communication as improving the content’s quality.

A systematic review indicates that YouTube videos on women’s health can be a useful tool not only for providing general information but also for offering direct visual aid and practical guidance [43]. This potential becomes even more significant for topics like breast engorgement, where mothers are seeking practical solutions. Therefore, although our study presents important findings specific to breast engorgement, as the literature also emphasizes, further research aimed at understanding how obstetric patients generally use YouTube as a health information source for the various problems they encounter during pregnancy and the postpartum period will fill the existing knowledge gap [53]. Moreover, it is well established that patients who have knowledge about the causes, pathophysiology, treatment, and prevention of a disease are more likely to actively participate in and comply with treatment or prevention procedures, ultimately improving health outcomes [54]. This highlights the importance of providing accurate, understandable, and accessible information through online health education materials and platforms [55].

The moderate quality and reliability observed in YouTube videos regarding breast engorgement are consistent with findings across other digital health platforms. Recent evaluations of AI chatbots, such as ChatGPT, Gemini, and Perplexity, have revealed that despite their innovative potential, these tools often provide medical information with low reliability and poor quality for conditions like low back pain (LBP) [56]. Similarly, a study investigating Turkish internet-based patient education materials for LBP found that the readability levels were significantly higher than the globally recommended sixth-grade level, falling into the ‘medium difficulty’ category according to the Atesman readability index [57]. When our findings are synthesized with these studies, a persistent ‘digital information barrier’ becomes evident. Whether through video (YouTube), AI interfaces, or text-based websites, health information frequently fails to meet the required quality, reliability, and readability standards. For a painful and acute condition like breast engorgement—which is a major factor in early weaning—it is imperative that digital content is tailored to the average education level of the target population and reviewed by expert teams. Our study reinforces the necessity for healthcare providers to not only produce evidence-based content but also ensure it is presented at an understandable readability level to effectively support maternal and public health.

Beyond the clinical accuracy of individual videos, our findings have broader implications for public health policy and digital health literacy. The identified ‘quality–popularity paradox’ suggests that health-seeking behavior in the postpartum period is significantly influenced by emotional engagement rather than scientific rigor. From a public health perspective, this necessitates a shift toward improving ‘Digital Health Literacy’ among new mothers, empowering them to critically evaluate online content [54]. Moreover, health policymakers and professional organizations should not only produce evidence-based content but also collaborate with digital platforms to implement more robust verification systems for maternal health information [4]. Integrating digital navigation skills into standard prenatal and postnatal education programs could serve as a proactive strategy to mitigate the risks associated with the consumption of unverified health advice on social media.

This study has several significant strengths. Firstly, it offers a substantial contribution to the field by analyzing YouTube content on a specific topic, breast engorgement, using standardized and widely accepted objective quality assessment tools such as DISCERN, JAMA, and GQS. The independent evaluations conducted by researchers from two different disciplines (an obstetrics and gynecology nurse and a midwife) enhance the reliability of the results. However, the study also has some limitations. The research has a cross-sectional design and is limited to specific keywords and English-language videos, which restricts the generalizability of the findings. Qualitative studies aimed at understanding viewers’ video preferences and perceptions of trust would guide healthcare professionals in developing more effective and wide-reaching health communication strategies. Another limitation of this study is the categorization of all healthcare providers into a single group. Since different types of providers possess varying scopes of practice and specialized expertise, future research should explicitly assess video content by specific provider types to provide more granular insights. Furthermore, the classification of healthcare professionals relied on self-identification within the videos. Due to the absence of a formal credential verification system on YouTube, some degree of misclassification may have occurred, which should be considered when interpreting the comparative results between professional and non-professional sources. In addition, it is important to consider the role of YouTube’s proprietary recommendation algorithms and geographic biases in shaping the search results analyzed in this study. Although we utilized ‘incognito mode’ to minimize personalized interference, search outcomes on YouTube are inherently influenced by the user’s geographic location (IP address) and the platform’s evolving metadata-driven ranking systems. This algorithmic curation means that mothers in different global regions may encounter different sets of information, which limits the direct reproducibility and universal generalizability of our findings. Future research should consider multi-regional or cross-linguistic analyses to account for these platform-specific biases. Finally, it must be acknowledged that YouTube metrics, including views, likes, and interaction rates, are highly dynamic and fluctuate continuously over time. The data presented in this study represent a specific snapshot captured between July 4 and August 6, 2025. Consequently, these values may vary in future assessments as the platform’s engagement and visibility of the content continue to evolve.

## Conclusion

This study aimed to evaluate the quality and reliability of YouTube videos concerning breast engorgement. The findings showed that although videos produced by healthcare professionals tend to be of higher quality based on DISCERN, JAMA, and GQS scores, this does not necessarily correspond with higher viewer interaction. This situation indicates that for a common and urgent issue like breast engorgement, mothers are at risk of turning to content that is of questionable accuracy but more attractively presented, instead of evidence-based, correct information. Our findings highlight a critical point for healthcare professionals: while digital platforms such as YouTube can contribute to the dissemination of health information, they cannot replace direct clinical care. Therefore, ensuring that mothers receive individualized support in managing conditions like breast engorgement should remain a primary focus. Additionally, strategies to reduce misinformation and the implementation of verification systems for health-related content on platforms like YouTube should be considered.

## Data Availability

The datasets used and analyzed during the current study are available from the corresponding author on reasonable request.
